# Seed weight and germination behavior of the submerged plant *Potamogeton pectinatus* in the arid zone of northwest China

**DOI:** 10.1002/ece3.1451

**Published:** 2015-03-06

**Authors:** Zhongqiang Li, Wei Lu, Lei Yang, Xianghong Kong, Xuwei Deng

**Affiliations:** 1 Hubei Collaborative Innovation Center for Green Transformation of Bio-Resources, Hubei Key Laboratory of Regional Development and Environmental Response, Faculty of Resource and Environment, Hubei UniversityWuhan, 430062, China; 2 Wuhan Research Academy of Environmental Protection SciencesWuhan, 430015, China

**Keywords:** Aquatic macrophytes, germination, maternal environment, seed fecundity, seed weight

## Abstract

Variation in seed weight is common within and among plant species, but few studies have attempted to document the pattern of seed weight and germination attributes for aquatic macrophytes at a large scale. This study examined within-species variation in seed weight and germination attributes and the effects of environmental factors on seed traits of the submerged plant *Potamogeton pectinatus* in the arid zone of northwest China. Our results showed that the average seed weight was 0.24 g per 100 seeds with a coefficient of variation (CV) of 28.4% among the eight *P. pectinatus* populations. The total germination fraction of seeds of *P. pectinatus* was relatively poor, less than 35% in seven *P. pectinatus* populations, and the lowest germination percentage found was only 2%. There were significant differences in seed weight, time to onset of germination, and total germination fraction among the eight different populations. Hierarchical partitioning analysis showed a strongly positive correlation between seed weight and water temperature and pH. Seed weight and the maternal environmental factors significantly affected both time to initiation of germination and total germination fraction. Our results suggest that (1) seed weight variation in *P. pectinatus* primarily is the result of temperature variation during fruit development; (2) relatively poor germination fraction suggests that seeds are relatively unimportant in the short-term survival of populations and that it may be another adaptive trait allowing plants to take place in the right place and at the right time, especially in harsh environment; and (3) variation in seed germination traits should be determined by local environmental and intrinsic factors that interact in a complex fashion.

## Introduction

Seed traits, including seed weight and germination, are critical characters of the life histories of higher plants (Harper 1977; Ellison 2001), and their importance to plant fitness is widely appreciated (Baskin and Baskin 1998; Tremayne and Richards 2000). Patterns of seed weight and germination and the respective controlling factors have been widely studied in the past few decades (Totland and Birks 1996; Tremayne and Richards 2000; Moles et al. 2007; Linkies et al. 2010).

Intraspecific studies of wild species are critical for gaining an understanding of the ecological significance of seed mass (Mazer 1989). Comparisons of seed weight within species may show more subtle variation between populations, and research results will provide clues on how environmental factors influence seed characteristics that allow us to consider adaptation of seed traits for plants with wide distributions. Traditionally, seed weight within a plant species is considered to be a remarkably constant characteristic (Harper et al. 1970; Harper 1977), and selection was predicted to produce one optimal seed weight within maternal plants (Smith and Fretwell 1974). However, numerous studies have demonstrated that seed weight within a species, or even an individual plant, can vary greatly (e.g., Hendrix 1984; Thompson 1984; Wolf et al. 1986; Wolfe 1995). Differences in seed weight within species have been noted for plant populations separated by great distances and between habitats with significant differences in temperature (McWilliams et al. 1968; Santamaría et al. 2003), photoperiod (Cook 1975), shade (Paz et al. 1999), and moisture (Dyuryagina 1975; Schimpf 1977). A number of studies, dealing with single species, reported increasing seed weight with higher altitude due to selection pressure (Holm 1994; Boulli et al. 2001; Pluess et al. 2005), but some others studies reported a decrease because of low temperature and short seasons (Totland and Birks 1996; Pluess et al. 2005) or no alteration (Ellison 2001) in seed weight with increasing altitude. These conflicting results highlight that further studies are needed to understand how geographical factors influence plant seed weight. Also, to our knowledge, few studies have attempted to document the pattern of seed weight and variation for aquatic macrophytes, despite aquatic higher plants being long recognized as suitable models for the study of phenotypic and physiological variations (e.g., Greulich et al. 2001; Lynn and Waldren 2001), due to wide distribution and limited genetic variation (Santamaría et al. 2003).

Germination is a critical stage in the life cycle of plants and often controls population dynamics (Harper et al. 1970; Harper 1977). In angiosperms, there is increasing evidence that environmental maternal effects affect both germination percentage (Li et al. 2005; Donohue 2009; Figueroa et al. 2010; Tielbörger and Petrü 2010; Cendán et al. 2013) and germination phenology (Lacey 1996; Cendán et al. 2013). Environmental factors that affect the mother plant, such as photoperiod and temperature, during seed maturation have also been found to influence seed germination of many species (e.g., Boyko et al. 2010; Figueroa et al. 2010). These factors may also influence the size of the seeds, which in turn may influence germination timing and success (Castro 2006), and within species, seed mass is often associated with probability or time of germination (e.g., McWilliams et al. 1968; Venable and Lawlor 1980; Hendrix 1984; Winn 1985). This is important because it has been shown that larger seeds have an advantage over smaller ones as a higher proportion of larger seeds will germinate and give rise to more vigorous seedlings (Baskin and Baskin 1998). However, little information on how environmental factor effects and seed weight are associated with the germination trait of macrophyte is available. Identifying the environmental mechanisms involved in the transmission of maternal environmental effects through the seeds is important to understand their ecological function and adaptive value (Cendán et al. 2013).

Wetland plant habitats are extremely varied and may be permanent or temporary, predictable or unpredictable, freshwater or saline, and have running or still water. The hydrologic regimen provides the overriding selective force, with climate as an important second factor (Leck and Brock 2000). Other factors such as temperature vary spatially and temporally, influencing seed character, germination processes and germination rates (Leck and Brock 2000). Aquatic plants include a variety of life forms and functional groups that are adapted to diverse wetland habitats (Santamaría 2002). Here, we hypothesized that the environmental conditions where the plant grows has a strong effect on the seed weight and interpopulation variation in seed germination traits should be determined by local environmental and intrinsic factors that interact in a complex fashion. Using a laboratory experiment to study the variation in seed weight of *P. pectinatus* and the potential effect of environmental factors of seed collection site on germination, we asked the following: (1) the variation in seed weight among a macrophytes population along large environmental gradient and the significance of such variation for germination behavior; (2) effects of environmental factors of seed collection site on seed germination behavior.

## Materials and Methods

### Study site

**Figure 1:**
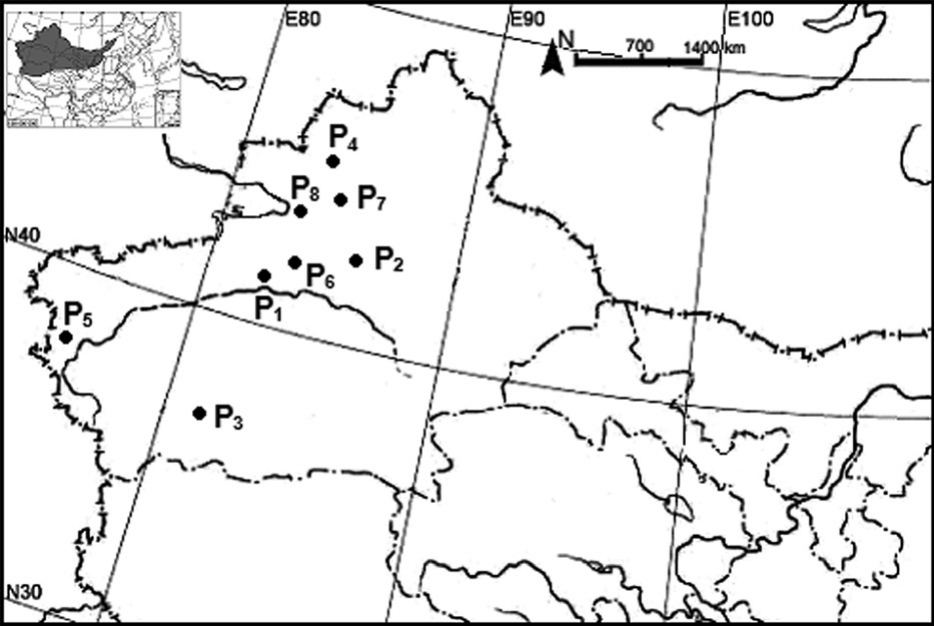
Map showing the collection sites of *Potamogeton pectinatus* seeds and its location in the arid zone of northwest China.

The arid zone (35°30–49°N, 73°–106°E) is a land-locked region located in northwestern China (Fig.1) and is surrounded by the Qinghai–Tibet Plateau and many high mountains. The climate is generally water limited, and steppe biomes are prevalent. The annual rainfall in the arid zone is less than 250 mm, with some areas receiving less than 100 mm annually, but the annual evaporative capability is above 2000 mm. The mean annual temperature is 2–6°C, with maximum monthly mean temperature over 28°C and minimum monthly mean temperature below −16°C, and the daily temperature fluctuates significantly (Feng et al. 1989).

### Study species and seed material

*Potamogeton pectinatus* L. is a perennial submerged aquatic macrophyte with a parvopotamid growth form. It has a cosmopolitan distribution and inhabits many different environments, for instance both in alkaline fresh and brackish water, in standing and running water, and in waters of different trophic status (Van Wijk 1988; Kantrud 1990). Furthermore, *P. pectinatus* is very common and often very abundant in many parts of its area of distribution (Van Wijk 1988). *P. pectinatus* reproduces by many different means, depending on habitat and environmental stress (Kantrud 1990), and its populations are primarily maintained interannually through subterranean tubers (specialized asexual propagules) with a minimal observed contribution from seeds (Van Wijk 1989). Instead, seeds might be important for dispersal and establishment after disturbances (Van Wijk 1989; Kantrud 1990).

*Potamogeton pectinatus* seeds material used in the germination experiments were collected from eight different sites located in the arid zone of northwest China (Fig.1) across from 36.8°N to 43.2°N in latitude, from 75.2°E to 86.5°E in longitude, and from 352 m to 3156 m in altitude between August and September in 2011. At each site, latitude, longitude, and altitude were recorded using GPS. We also measured the pH, water temperature, conductivity, and salinity of each water body using an HORIBA U-10 Water Quality Checker (HORIBA, Kyoto, Japan). For each population, *P*.* pectinatus* grew well and its coverage was above 85%. For seed material, only drupelets attached to plants were used, in order to be certain of age and origin. After collection, the material was stored for some time in darkened glass jars in tap water under controlled temperature (about 25 ± 5°C), allowing the seed to ripen, when still attached to the plants (Van Wijk 1988). After sometime, the ripe seed detached and were collected and then air-dried at room temperature. Then, the seed materials were transported to the laboratory stored in dark refrigerator at 4°C until germination studies were initiated. For this population-level study, all seeds from each site were collected from each of more than ten plants and thoroughly mixed to minimize effects of single parental plant on germination.

### Controlled germination trials

To avoid confounding with numerous factors that control germination in the field (e.g., soil type, temperature, pH, salinity; see Tielbörger and Petrü 2010), we carried out germination trials in a climate chambers at the Hubei University in Wuhan, China, from 26 March 2012 to 13 June 2012 in the laboratory. Before the germination experiment, the seeds were allowed to air-dry to a constant mass at room temperature. One hundred air-dried seeds randomly chosen from each mixed collections were weighed, and three replicates were employed. Also, air-dried seeds were used in the germination trials. For germination, seeds of each population were chosen at random from mixed collection regardless of their size because variation of seed weight in the same population is very small. Three replicates of 50 seeds were tested for each population. Fifty seeds were put into 9-cm diameter Petri dishes on a double layer of a filter paper and moistened with sterile distilled water. All 24 Petri dishes were placed in the same climate chamber. We exposed the seeds to a temperature range of 25°C day/15°C night, and the relative humidity was kept at 85%. A light regime of 12-h light and 12-h dark was maintained with Philips TL tubes type 33, providing 70 *μ*Em^−2^s^−1^. Distilled water was added to each dish every day to prevent desiccation. Seed germination was monitored every day. A seed was considered to have germinated when the radicle was 1 mm in length. Time (days) to first-observed germination and total number of seeds germinated after 79 days were recorded. Germinated seeds were discarded after recording. Total germination fraction was calculated by the number of seeds germinated during experiment in relation to total initial seed number 50 seeds (in %).

### Data analysis

To explore the influence of climate on seed weight, we analyzed the relationship between seed weight and mean annual temperature and mean annual precipitation. The climate data used in this study were calculated based on linear models using latitude, longitude, and altitude as variables from 50-year averaged temperature and precipitation records (1951–2000) at 680 well-distributed climate stations across China (Fang et al. 2001; Piao et al. 2003).

Statistical analysis was carried out using SPSS 17.0 software (SPSS Inc., Chicago, IL, USA). Analysis of variance was applied to determine the statistical significance of the differences of seed weight and time to onset of germination across population (*P *<* *0.05). Before performing one-way ANOVA, all data were tested for normality and homogeneity. Nonhomogeneity data were transformed (1/x) to obtain homogeneity. The differences in total germination fraction across population were tested by a *chi-square* test. Means and standard deviation (SD) were reported in the tables and figures are calculated in the basis of untransformed data.

Because of the colinearity of climatic variables and environmental factors, we directly estimated their independent contributions of most significant climate and environmental variables to variation in the seed weight and germination traits using hierarchical partitioning analysis (R 2.11.1, the R Foundation for Statistical Computing) Vienna, Austria with a general linear model and bootstrapping (MacNally and Walsh 2004; Zhou et al. 2013). Where seed germination traits were significantly correlated with climate and environmental variables, a partial correlation analysis was carried out to clarify the relationship between response variable and climate and environmental by controlling for seed weight. Also, univariate linear regression analyses were applied to examine the effects of seed weight on seed germination traits.

## Results

### Variability in seed weight and seed germination traits

There was great variation in mean seed weight per 100 seeds in the eight *P. pectinatus* populations sampled, ranging from 0.16 ± 0.002 g to 0.35 ± 0.001 g, and the heaviest seeds occurred in P_1_, and the lightest seed occurred in P_5_ (Table1). The mean weight per 100 seeds was 0.24 g and showed a 2.5-fold range overall, with a coefficient of variation (CV) of 28.44%. Results of the ANOVA showed that there were significant differences in seed weight among the eight different populations (*P *<* *0.01) (Table1).

**Table 1:** Mean (mean ± SD) and results of the ANOVA (*F*-value) of seed weight per 100 seeds and time to germination and of the chi-square test of total germination fraction (*n* = 3) of eight different *Potamogeton pectinatus* populations in the arid zone of the Northwest China

	Seed weight per 100 seeds (g)	Time to germination (days)	Total germination fraction (%)
P_1_	0.35 ± 0.001	10.0 ± 3.0	58.67 ± 1.15
P_2_	0.17 ± 0.006	18.0 ± 0.0	2.0 ± 0.00
P_3_	0.20 ± 0.005	21.0 ± 0.0	14.67 ± 3.05
P_4_	0.20 ± 0.008	27.00 ± 2.3	3.33 ± 2.31
P_5_	0.16 ± 0.002	13.0 ± 0.0	32.67 ± 6.11
P_6_	0.27 ± 0.01	16.0 ± 0.0	28.67 ± 6.43
P_7_	0.29 ± 0.02	9.0 ± 3.46	16.0 ± 2.0
P_8_	0.31 ± 0.008	12.0 ± 1.73	30.67 ± 5.77
*F*	142.4***	48.8***	–
*χ*^2^	–	–	21.99**

**P <* *0.01; ***P <* *0.001.

**Figure 2:**
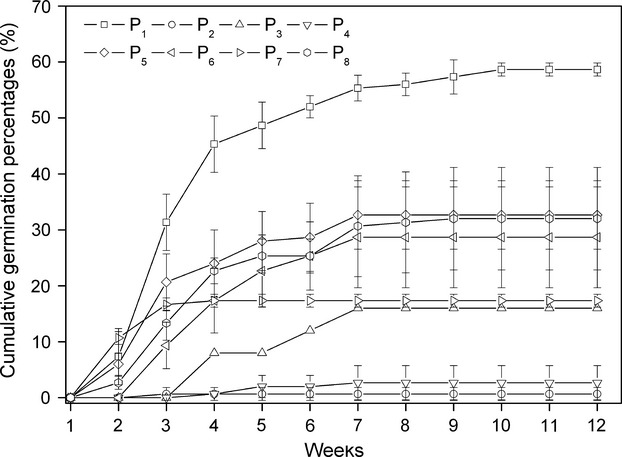
Total germination fraction (Mean ± SE) of 50 seeds of *Potamogeton pectinatus* incubated for 12 weeks in the climate chambers.

For seed germination traits under controlled experimental conditions, the fastest time to start of germination observed in P_7_ (9.0 ± 3.46 days) and P_1_ (10.0 ± 3.0 days) and the slowest time to start of germination observed in P_4_ (27.67 ± 2.3 days) (Table1). The total germination fraction of seeds of *P. pectinatus* was relatively poor (Table1, Fig.2), less than 35% in seven *P. pectinatus* populations. Among eight *P. pectinatus* populations, the highest and lowest total germination fractions were 58.67 ± 1.15 found in P_1_ and 2.0 ± 0.00 found in P_2_, respectively (Table1). ANOVA and the chi-square test revealed that significant difference occurred among eight populations in time to start of germination (*P *<* *0.01) and total germination fractions (*P *<* *0.01), respectively (Table1).

### Effects of climate and environmental factors on seed weight and germination behavior

Hierarchical partitioning analysis of the effects of the nine variables revealed that pH and water temperature had the significant effect on seed weight. Time to germination was influenced significantly by altitude and pH. And altitude, longitude, and water temperature markedly influenced total germination fraction (Table2). However, a partial correlation analysis controlling for the effect of seed weight indicated that among climate and environmental factors above mentioned, only altitude had a significant effect on the time to initiation of germination and the total germination fraction under controlled laboratory conditions (Table3).

**Table 2:** Hierarchical partitioning of the independent effects of nine climate and environmental variables on three traits of seed in eight different *Potamogeton pectinatus* populations

	Fraction of regression relationship (%) attributable independently to environmental variables for each trait		
	Seed weight	Time to germination	Total germination fraction
Latitude			
Longitude			13.97
Altitude		13.51	14.77
Conductivity			
Salinity			
Water temperature	19.39		16.75
pH	23.69	27.84	
Mean annual temperature			
Mean annual precipitation			

Nonsignificant contributions (*P *>* *0.05) are omitted. Probabilities are based on bootstrapping with 1000 repetitions.

**Table 3:** Partial correlation coefficients (*r*) between time to germination and total germination fraction and longitude, altitude, pH, and water temperature controlling for the effects of seed weight

	Longitude	Altitude	pH	Water temperature
Time to germination	0.36	−0.64**	−0.30	−0.28
Total germination fraction	–	0.83***	−0.13	–

**P <* *0.01; ***P <* *0.001.

### Seed germination traits response to seed weight

**Figure 3:**
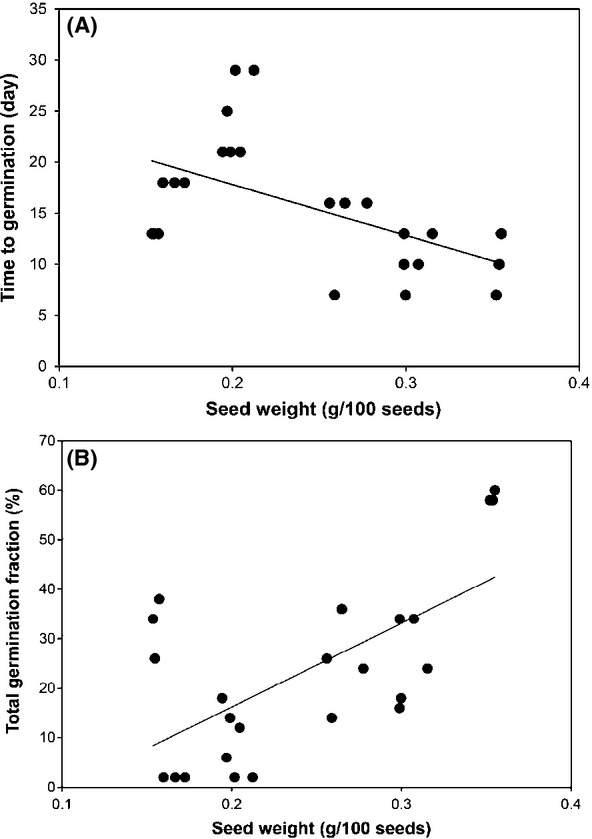
Scatter plots showing the relationships among seed weight and time to germination and total germination fraction of eight *Potamogeton pectinatus* population. (A) Seed weight and time to germination (*R*^2^* *=* *0.30, *P *=* *0.006, *n* = 24) and (B) seed weight and total germination fraction (*R*^2^* *=* *0.42, *P *=* *0.001, *n* = 24) are displayed.

Time to start of germination significantly covaried with seed weight (*P *=* *0.006), with heavier seeds germinating earlier (Fig.3). Seed total germination fractions were also significantly associated with seed weight in different *P. pectinatus* populations. Univariate linear regression analysis indicated that seed weight values were highly positively correlated with seed total germination fractions (*P *=* *0.001; Fig.3).

## Discussion

Comparative analyses of seed weight and seed germination traits have been especially useful in illustrating not only the broad range of seed traits that occur within taxa, but also the environmental constraints that limit variability in characters (Ellison 2001). In the present study, our results clearly showed that there were significant differences in seed weight among populations (Table1). Thompson (1984) noted that CVs over 5% are often considered “large” in biological systems and by the 5% criterion, *P*. *pectinatus* in the study exhibits “large” CVs, indicating that within-species variation in seed weight is considerable. Such interpopulation variation in average seed weight may arise if the environmental conditions experienced by the plants differ among population localities and/or if there are genetic differences in traits directly related to seed weight (Totland and Birks 1996). Seed weight is known to be strongly influenced by the environmental factors where plants grow (Linkies et al. 2010). External factors that affect the growth environment can influence the ability of the plant to resource embryos and have been shown to cause variation in seed weight (Tremayne and Richards 2000). Many factors might contribute to variation in seed weight within *P*. *pectinatus* populations. (1) Differences between populations with respect to altitude, latitude, and longitude may imply that different populations experience different climatic conditions. (2) Abiotic factors where plant grew could contribute to variation in resource availability and reproductive performance. A number of studies, dealing with single species, reported larger seed at higher altitude, probably because of selection pressure (Holm 1994; Boulli et al. 2001; Pluess et al. 2005). Other studies suggested that high-altitude species generally produce smaller seeds than their lower altitude congeners, possibly due to a shorter growing season and low temperature (Totland and Birks 1996). However, our findings indicate that in macroscale, seed weight does not directly correlate with geographical factors (latitude, longitude, and altitude) or meteorological factors (MAT and MAP) (Table2). It is notable that seed weight directly correlated with water temperature of habitat, despite the weak relationships found between seed weight and MAT (Table2). This may be because water temperature, rather than air temperature, can directly affect sediment temperature where aquatic plants grow. Moreover, at mid-to-high latitudes, the mean and variation in water temperatures are far more moderate than in air (Luning 1993; Reynolds and Smith 1995), leading to increasing disparity between air and water temperature in mean values and seasonal amplitude with increasing latitude. So, for aquatic plants, water body temperature is more important than MAT as an influence on plant physiology and reproduction. In higher water temperature where plant grew, the species generally produce bigger seeds than their lower water temperature congeners, possibly due to a longer growing season and increasing the speed of seed maturation (Totland and Birks 1996). Moreover, if the green, developing achenes of *P. pectinatus* are photosynthetic (Bazzaz et al. 1979), the lower water temperatures of habitat where plant grew may reduce the photosynthesis rate, and thereby reducing the mean seed weight in these populations. Also, our results indicated that seed weight is strongly correlated with pH of water body, suggesting that pH is one of the most important abiotic factors which result in interpopulation variation in seed weight. Previous study demonstrates that *P. pectinatus* can tolerate alkalinity but grow poorly in acidic water (Kantrud 1990). It is clear that variation in seed weight results from variation in *P. pectinatus* growth, development, and metabolism in different population driven by different local abiotic factors. These factors might influence plant vigor so that seed weight might be associated with such attributes as plant size, flower number, flower size, fruit number, and seed number (Wolfe 1995).

In our study, total germination fraction of *P. pectinatus* under control experimental condition is very low (Fig.2). The results of the germination experiments with achenes coincide with those found in control experiments and in the natural habitats (Van Wijk 1988, 1989; Kantrud 1990). Low total germination fraction among *P. pectinatus* populations indicated that seeds are relatively unimportant in the short-term survival of populations (Van Wijk 1989; Kantrud 1990). Thus, the importance of seed for *P. pectinatus* in the yearly survival is probably only limited, as this species reproduction is mainly vegetative (Van Wijk 1989). The main function of *P. pectinatus* seed is most likely the dispersal of the species and the long-term survival (Kantrud 1990), playing important roles in colonization at the edges of population (as a result of sediment movement) (Terrados 1993), serving to establish new genotypes in an existing population and providing a mechanism to re-establish populations after disappearance (Van Wijk 1989).

Our results revealed that environmental factors where *P. pectinatus* grew had a strong influence on both the time to onset of germination and the total germination fractions (Tables2, and 3). Seeds from higher altitude sites germinated earlier, and the proportion of seeds that remained ungerminated at the end of the experiment was lower at higher altitude. Some of this variation can be of genetic origin, but much of it is known to be environmental, that is, caused by the local conditions under which the seed matured. Evidences suggest that maternal factors, such as position of the seed in the fruit of the mother plant during seed maturation, markedly influence the germinability of seeds (Baskin and Baskin 1998; Alboresi et al. 2005; Donohue 2009; Figueroa et al. 2010; Tielbörger and Petrü 2010; Cendán et al. 2013). The differences in the reaction of *P. pectinatus* to different environmental factors where plant grew suggest that there is strong selection pressure for variation rather than uniformity in germination requirements within populations of *P. pectinatus*. One possible explanation for germinated earlier at higher altitude is that seeds of the higher altitude *P. pectinatus* population had a lower cumulative temperature requirement for germination than those of the lower altitude population. Higher total germination fraction at higher altitude may be another adaptive trait allowing plants to take place in the right place and at the right time, especially in harsh environment because more sexual reproduction could provide more genetic diversity and increase fitness to unpredictable and harsh environment (Barrat-Segretain 1996). It is well known that environmental conditions where the mother plant grows may largely influence the size of the seeds, which in turn may influence seed germination traits (Castro 2006). As has been found for a numbers of species (Baskin and Baskin 1998), the larger seeds had a significantly higher germination percentage than the smaller ones. In our case, the germination attributes significantly covaried with seed weight, with bigger seeds tending to germinate earlier and in greater number, indicating that the variations in germination attributes of the studied species appear to be based partly on the effects of maternal environmental factors, but also on intrinsic effects of seed weight. This result may be explained by that small seeds probably have a reduced vigor or viability due to marginal environments.

In summary, our results support our hypotheses that the environmental conditions where the mother plant grows may largely influence seed weight, which in turn may influence seed vigor or viability. Variation in seed weight of *P. pectinatus* primarily may be the result of temperature variation during fruit development. Relatively poor germination fraction of *P. pectinatus* indicated that seeds are relatively unimportant in the short-term survival of populations and it may be another adaptive trait allowing plants to take place in the right place and at the right time, especially in harsh environment. Seed weight had significant influence on seed germination time and total germination fraction. Besides maternal intrinsic genetic effects, the maternal environmental factors significantly affected both time to initiation of germination and total germination fraction, indicating that genetic variation in the sensitivity to the maternal environment in this *Potamogeton* species does exist. Variation in seed germination traits should be determined by local environmental and intrinsic factors that interact in a complex fashion. Thus, further study is recommended to separate and quantify the influence of the maternal genotype, the maternal environment, and their interaction.

## Appendix

**Table A1:** Geographical and water environmental characters and estimated data (e.g., mean annual temperature and mean annual precipitation) of each sampling collection site

Site	Lat	Long	Alt (m)	Cond (mS/cm)	Sal (%)	pH	Wt (°C)	MAT (°C)	MAP (mm)
P1	41.09°	82.59°	964	3.77	0.18	9.0	24.4	11.18	72.81
P2	41.77°	86.49°	870	6.12	0.33	7.8	21.1	10.68	65.81
P3	36.89°	81.62°	1373	3.9	0.2	8.6	21.6	12.18	16.39
P4	44.61°	82.84°	352	0.44	0.01	8.2	18.1	9.69	137.26
P5	37.78°	75.24°	3156	0.40	0.01	8.2	12.5	1.45	172.72
P6	41.67°	82.79°	963	0.72	0.03	8.0	17.8	10.69	83.88
P7	43.52°	83.26°	831	1.13	0.05	9.3	18.3	8.29	135.19
P8	43.18°	82.59°	318	13.7	0.79	9.7	27.3	11.46	103.08

Lat, latitude; Long, longitude; Alt, altitude; Cond, conductivity; Sal, salinity; Wt, water temperature; MAT, mean annual temperature; MAP, mean annual precipitation.
